# Advances in Near-Infrared BODIPY Photosensitizers: Design Strategies and Applications in Photodynamic and Photothermal Therapy

**DOI:** 10.3390/ph19010053

**Published:** 2025-12-26

**Authors:** Dorota Bartusik-Aebisher, Kacper Rogóż, Gabriela Henrykowska, David Aebisher

**Affiliations:** 1 Department of Biochemistry and General Chemistry, Collegium Medicum, Faculty of Medicine, University of Rzeszów, 35-310 Rzeszów, Poland; dbartusikaebisher@ur.edu.pl; 2 English Division Science Club, Collegium Medicum, Faculty of Medicine, University of Rzeszów, 35-310 Rzeszów, Poland; kr117626@stud.ur.edu.pl; 3 Department of Epidemiology and Public Health, Faculty of Medicine, Medical University of Lodz, 90-419 Lodz, Poland; 4 Department of Photomedicine and Physical Chemistry, Collegium Medicum, Faculty of Medicine, University of Rzeszów, 35-310 Rzeszów, Poland

**Keywords:** photodynamic therapy, PDT, photosensitizers, BODIPY, near-infrared, photothermal therapy, PTT

## Abstract

**Background/Objectives:** Boron-dipyrromethene (BODIPY) derivatives are a superior class of fluorophores prized for their exceptional photostability and tunable photophysical properties. While ideal for imaging, their translation to photodynamic therapy (PDT) has been hampered by excitation in the visible range, leading to poor tissue penetration. To overcome this, intense research has focused on developing near-infrared (NIR)-absorbing BODIPY photosensitizers (PS). This review aims to systematically summarize the hierarchical design strategies, from molecular engineering to advanced nanoplatform construction, that underpin the recent progress of NIR-BODIPY PS in therapeutic applications. **Methods:** We conducted a comprehensive literature review using PubMed, Scopus, and Web of Science databases. The search focused on keywords such as “BODIPY”, “aza-BODIPY”, “near-infrared”, “photodynamic therapy”, “photothermal therapy”, “nanocarriers”, “hypoxia”, “immuno-phototherapy”, and “antibacterial.” This review analyzes key studies describing molecular design, chemical modification strategies (e.g., heavy-atom effect, π-extension), nanoplatform formulation, and therapeutic applications in vitro and in vivo. **Results:** Our analysis reveals a clear progression in design complexity. At the molecular level, we summarize strategies to enhance selectivity, including active targeting, designing “smart” PS responsive to the tumor microenvironment (TME) (e.g., hypoxia or low pH), and precise subcellular localization (e.g., mitochondria, lysosomes). We then detail the core chemical strategies for achieving NIR absorption and high singlet oxygen yield, including π-extension, the internal heavy-atom effect, and heavy-atom-free mechanisms (e.g., dimerization). The main body of the review categorizes the evolution of advanced theranostic nanoplatforms, including targeted systems, stimuli-responsive ‘smart’ systems, photo-immunotherapy (PIT) platforms inducing immunogenic cell death (ICD), hypoxia-overcoming systems, and synergistic chemo-phototherapy carriers. Finally, we highlight emerging applications beyond oncology, focusing on the use of NIR-BODIPY PS for antibacterial therapy and biofilm eradication. **Conclusions:** NIR-BODIPY photosensitizers are a highly versatile and powerful class of theranostic agents. The field is rapidly moving from simple molecules to sophisticated, multifunctional nanoplatforms designed to overcome key clinical hurdles like hypoxia, poor selectivity, and drug resistance. While challenges in scalability and clinical translation remain, the rational design strategies and expanding applications, including in infectious diseases, confirm that NIR-BODIPY derivatives will be foundational to the next generation of precision photomedicine.

## 1. Introduction

Despite significant advances in conventional therapies such as chemotherapy, radiotherapy, and surgery, modern oncology still faces fundamental problems, including systemic toxicity of treatment, lack of selectivity, and the development of multidrug resistance (MDR) [1,2]. In response to these challenges, therapeutic strategies based on external stimuli are being intensively developed, among which light-activated therapies—photodynamic therapy (PDT) and photothermal therapy (PTT)—have gained prominence due to their unparalleled spatial and temporal control, allowing for precise tumor ablation with minimal damage to healthy tissues [3,4].

PDT relies on a photosensitizer (PS) which, upon light excitation and intersystem crossing (ISC), generates cytotoxic species via Type I (radicals) or Type II (singlet oxygen, ^1^O_2_) mechanisms [5,6,7]. Conversely, PTT agents convert absorbed photon energy directly into local hyperthermia through non-radiative relaxation, causing thermal denaturation of proteins [8,9].

**Figure 1:**
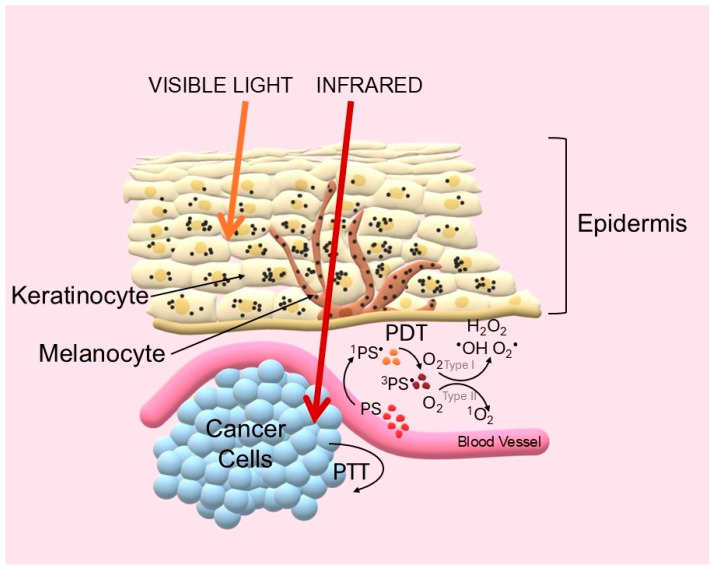
Schematic diagram of photodynamic (PDT) and photothermal (PTT) therapy mechanisms and biological optical windows. Light Penetration: Visible light is strongly attenuated by endogenous chromophores (e.g., hemoglobin, melanin) and scattering. In contrast, NIR light, particularly in the NIR-I and NIR-II windows, minimizes these interactions, allowing for deep tissue penetration. PDT Mechanism: Upon NIR absorption, the photosensitizer transitions to an excited singlet state (^1^PS•). It can undergo intersystem crossing (ISC) to a long-lived triplet state (^3^PS•). From this state, the PS generates cytotoxicity via two pathways: electron transfer producing radicals (Type I) or energy transfer to molecular oxygen generating toxic singlet oxygen (^1^O_2_) (Type II). PTT Mechanism: Alternatively, the excited PS returns to the ground state via non-radiative vibrational relaxation, converting absorbed energy directly into local hyperthermia for thermal ablation (Created by the authors).

Historically, the effectiveness of phototherapy has been limited by the poor penetration of visible light into tissues due to absorption by endogenous chromophores such as hemoglobin and melanin [10]. A breakthrough came with the use of near-infrared (NIR) photons operating in the so-called biological (or theranostic) windows. The first window (NIR-I, 700–950 nm), and especially the second (NIR-II, 1000–1700 nm), are characterized by drastically reduced scattering and absorption, allowing light to penetrate to a depth of several centimeters [11,12]. The challenge was therefore to design a new generation of photosensitizers that would be active in this range [13]. The key to the success of phototherapy lies in the precise use of light energy. These fundamental principles are illustrated in Figure 1.

Among the many classes of dyes, BODIPY (boron-dipyrromethene) derivatives have emerged as a platform with almost unlimited adaptability. These molecules, although in their basic form they absorb in the visible range and are characterized by high fluorescence quantum yield (making them ideal for imaging but not for therapy), are extremely susceptible to chemical modification. Rational molecular design, such as extending the π system of the core, the internal heavy-atom effect or heavy-atom-free strategies, has allowed their properties to be “switched” from fluorophores to highly efficient photosensitizers and photothermal converters, with absorption precisely tuned to the NIR range [14].

This review focuses on the latest advances in NIR-absorbing BODIPY photosensitizers. We discuss molecular design strategies for enhancing selectivity (TME-responsiveness, subcellular targeting) and optimizing photophysical properties. Furthermore, we analyze the evolution of these molecules into advanced theranostic nanoplatforms capable of overcoming biological barriers, and highlight emerging applications in antibacterial therapy and biofilm control.

## 2. Materials and Methods

This article provides a narrative review of the literature on advances in the design and biomedical applications of near-infrared absorbing BODIPY photosensitizers. In the first stage, a database search was performed using the query string: BODIPY AND (“near infrared” OR “near-infrared”) AND (PDT OR “photodynamic therapy”), which yielded a total of 614 results: 106 in PubMed, 173 in Scopus, and 335 in Web of Science. After removing duplicates, 175 unique publications were selected for preliminary analysis, of which 73 articles were cited after reviewing the abstracts and full texts.

**Figure 2:**
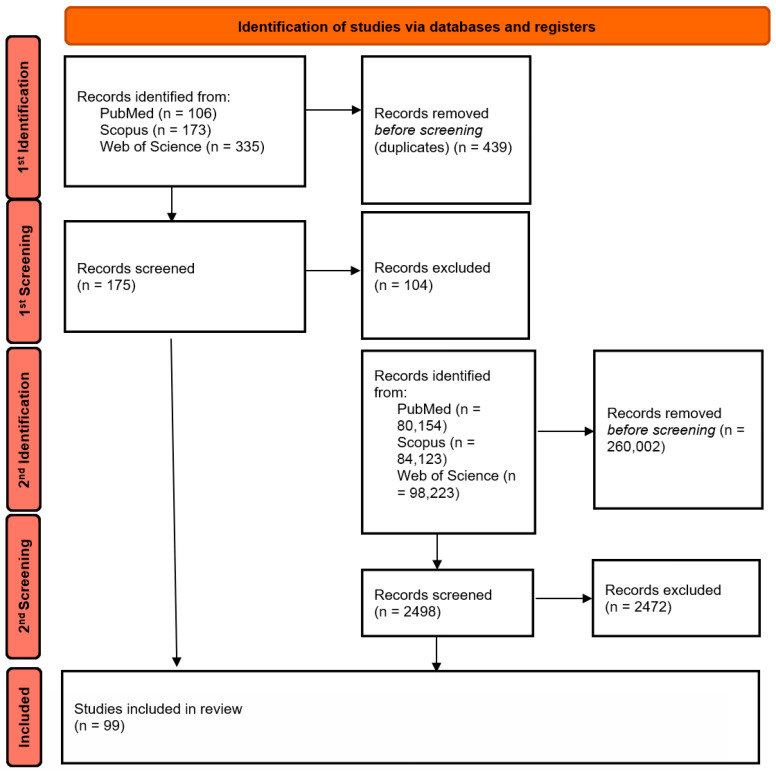
Literature selection process (PRISMA-style adapted flowchart for narrative review). The letter “*n*” stands for the word “number” (Created by the authors).

In the second step, the search was expanded to include related and more specific terms, such as: “aza-BODIPY”, “photothermal therapy”, “nanocarriers”, “hypoxia”, “immuno-phototherapy”, “antibacterial”, and similar terms, in order to capture reports not included in the first stage but relevant to specific nanotechnological and antibacterial applications. The literature search covered the period from the inception of the databases up to October 2025. Inclusion criteria comprised: (i) peer-reviewed original research and review articles; (ii) publications in the English language; (iii) studies focusing specifically on BODIPY derivatives with absorption/emission in the NIR range (>650 nm) or activated via two-photon excitation; (iv) papers reporting biological applications (PDT, PTT, imaging, antibacterial). Exclusion criteria included: (i) conference abstracts, editorials, and letters; (ii) studies focusing solely on visible-light BODIPY without NIR modification strategies; (iii) purely synthetic papers lacking photophysical or biological evaluation. After applying the inclusion criteria, 99 publications were ultimately selected for detailed analysis. The literature selection process is presented in Figure 2 in the form of a PRISMA-style literature flow diagram, adapted to a narrative review.

## 3. Strategies for Designing BODIPY Photosensitizers in Cancer Therapy

In recent years, there has been intensive development of applications for compounds from the BODIPY (boron-dipyrromethene) family in cancer diagnosis and therapy. Thanks to their high molar absorption, photochemical stability, and possibility of structural modification, these compounds represent a promising platform for cancer phototherapy. As described in a review article on the synergistic applications of BODIPY in oncology, their derivatives can act as photosensitizers in photodynamic therapy (PDT), photothermal converters in photothermal therapy (PTT), and contrast agents in fluorescence and photoacoustic imaging [15,16]. In recent years, numerous small-molecule NIR-II photosensitizers have been developed, including BODIPY derivatives. Compared to NIR-I, the use of NIR-II dyes improves the precision of pathological lesion localization and enables photomediated therapy in deeper tissues, which is crucial in theranostics [17].

The key challenge in photodynamic therapy is to ensure selectivity of action. Structural modifications of the BODIPY core, including substitutions at the meso, α, and β positions and modification of the BF_2_ group, allow for precise adjustment of photophysical and phototoxic properties, as well as targeting of the photosensitizer to specific cellular organelles, which increases the effectiveness of PDT [18]. An example of active targeting is aza-BODIPY conjugates with active molecular targeting, which show increased uptake by melanoma cells. Studies conducted on compounds **1a** and **1b** have shown that the use of targeting ligands significantly increases the accumulation of the photosensitizer in tumor tissue and enables intracellular internalization through the mechanism of clathrin-mediated endocytosis. Both compounds were characterized by absorption in the NIR range (λmax ~680 nm) and high singlet oxygen generation efficiency, which translated into a strong phototoxic effect. In the mouse melanoma model, compound **1b** showed the highest therapeutic efficacy, leading to almost complete inhibition of tumor growth after irradiation [19]. Another approach utilizing the tumor microenvironment is the design of molecules activated by low pH. The developed pH-activated BODIPY photosensitizers absorbing in the NIR range are an example of an advanced therapeutic platform that enables precise differentiation of tumor cells based on two independent selection mechanisms: specific recognition of biotin or αvβ3 integrin receptors and the acidic tumor microenvironment. The use of protonatable amino groups allows for control of PET efficiency, leading to up to a fourfold increase in singlet oxygen generation under acidic conditions. In in vitro studies, these compounds have shown high phototoxicity towards cancer cells with no dark toxicity and minimal activity towards normal cells [20].

The literature increasingly emphasizes that the effectiveness of classic photodynamic therapy (PDT) is limited by tumor microenvironment hypoxia, which reduces the efficiency of reactive oxygen species (ROS) generation. The use of oxygen-independent type I photosensitizers has proven to be a solution to this problem. An example of this approach is the BODIPY-based auto-deactivating PDT strategy described by Zhang et al. The authors designed a cationic diiodo-BODIPY derivative (PyBDP) and its mitochondriotropic version TBDP, which is characterized by a redox-dependent activation mechanism. TBDP uses intracellular NADH as an electron donor, inducing disruption of the mitochondrial respiratory chain in cancer cells even under hypoxic conditions. Importantly, after therapy, the photosensitizer undergoes irreversible reduction to an inactive form (Leuco-TBDP), which minimizes the risk of post-treatment phototoxicity. The compound exhibits exceptionally high phototoxicity (IC_50_ = 0.095 μM) and low toxicity in the dark, indicating its potential in the treatment of hypoxia-resistant cancers [21].

In order to maximize the cytotoxic effect, modern photosensitizers are designed to accumulate in key cellular organelles. An example is targeting mitochondria, as demonstrated for the compound BDP2. BDP2 has been shown to not only efficiently generate reactive oxygen species under light exposure, but also to modulate mitochondrial dynamics by downregulating the expression of the fusion proteins MFN2 and OPA1. The oxidative stress induced in this way leads to fission-fusion imbalance, changes in mitochondrial morphology, and the initiation of apoptosis. Thanks to these properties, BDP2 effectively inhibits the process of choroidal neovascularization, providing an example of an advanced NIR-PDT platform with targeted mitochondrial action [22]. Although the study concerned a model of choroidal neovascularization, the same mechanism (disruption of mitochondrial dynamics and induction of apoptosis) has a direct translation into cancer therapies. Lysosomes are an equally promising target. The newly developed photosensitizer T2BDP-lyso exhibits selective accumulation in lysosomes (Pearson = 0.89), which, combined with low toxicity in the dark and high phototoxicity, enables effective eradication of A549 cells after irradiation with NIR light. The large two-photon absorption cross section (138.7 GM) additionally allows the compound to be used in two-photon imaging in vivo, which reinforces its potential as a new generation photosensitizer with dual diagnostic and therapeutic functions [23]. Another target, specific to certain types of cancer, are lipid droplets. The 6DBF2 photosensitizer represents an advanced class of compounds capable of two-photon photodynamic activation in the near-infrared range. It enables effective therapy in deep-seated tissues. Its spontaneous, specific accumulation in lipid droplets without the need for additional functionalization increases the selectivity of its action against cancer cells. In vitro and in vivo models have shown clear ablation of cancer cells under the influence of two-photon NIR irradiation, confirming the high therapeutic potential of this photosensitizer [24].

A separate strategy, allowing for deeper penetration and high precision of therapy, is the design of compounds capable of two-photon absorption (TPA) in the NIR-II window. This property is exhibited, among others, by the aforementioned T2BDP-lyso compounds [23] and 6DBF2 [24], characterized by a large TPA cross section. Further development of this concept is represented by new mono- and dimeric derivatives of distyryl-BODIPY, exhibiting emission in the NIR-I range and the ability to undergo two-photon excitation in the NIR-II window. For the first time, the use of a femtosecond Cr:Forsterite laser (1250 nm) allowed for the effective excitation of these structures through two-photon absorption, with a clear cooperative effect observed in dimers, leading to an increase in the TPA cross section. These properties make distyryl-BODIPY promising candidates for deep tissue bioimaging and potential applications in photodynamic cancer therapy, especially in procedures requiring limited phototoxicity and high spatial resolution [25].

## 4. Chemical Strategies and Structural Modifications in PS BODIPY Design

Recent advances in the field of aza-BODIPY dyes have focused on introducing new substituents to improve their photophysical and therapeutic properties. Of particular importance are structural modifications of the BODIPY core leading to the expansion of the conjugated π system and the introduction of substituents that increase singlet oxygen generation efficiency and shift absorption towards the NIR range. This allows for more effective light penetration into tissues and improves the selectivity of anticancer therapy [15].

The basic strategy for achieving absorption in the NIR range is to expand the conjugated π system of the BODIPY core. One method is to expand symmetric and asymmetric aza-BODIPYs with aryl groups through the Suzuki–Miyaura reaction. The introduction of electron-donating substituents, such as 4-methoxyphenyl or N,N-diphenylamino-biphenyl, resulted in significant bathochromic shifts in the absorption and emission bands towards NIR and an increase in the efficiency of singlet oxygen generation (ΦΔ up to 0.74). In particular, the AA1 and CC1 derivatives exhibited intense absorption bands in the 630–750 nm range and high photochemical stability [26].

Another approach is the fusion of heterocyclic rings, as exemplified by the family of bisbenzothieno[b]-fused BODIPYs (BTB), in which the integration of two benzothiophene units leads to significant structural stiffening and enhanced electron coupling. BTB compounds are characterized by intense absorption in the range of 643–665 nm, high molar extinction coefficients, and effective singlet oxygen generation, reaching values of Φ(^1^O_2_) up to 0.41 without the need for heavy atoms. The observed properties result from a reduction in the energy gap between the ^1^PS• and (^3^PS•) states and increased spin–orbit coupling, which promotes efficient inter-system transitions. In particular, Me_2_NPh-substituted BTB showed outstanding photocytotoxicity (IC_50_ = 83.5 nM in HeLa cells) [27]. A similar effect of core stiffening and conjugation extension was used in thieno-pyrrole-conjugated BODIPY derivatives. They represent a new class of photosensitizers capable of efficient singlet oxygen generation without the involvement of heavy halogen atoms. Molecules from the SBDPiR series, including SBDPiR690 and SBDPiR688, exhibit favorable photophysical properties resulting from core stiffening and the presence of electron-withdrawing substituents, which promote efficient intersystem crossings. SBDPiR688, in particular, is characterized by a high extinction coefficient and balanced emission and generation of ^1^O_2_, enabling simultaneous NIR imaging and effective photodynamic therapy in vivo [28].

Equally important as NIR absorption is ensuring high singlet oxygen generation efficiency. A classic method of increasing the inter-system crossing (ISC) probability is to use the heavy atom effect. An example is the stepwise bromination of AmBXI molecules, which significantly increased spin–orbit coupling, resulting in a systematic increase in singlet oxygen generation efficiency. AmBXI dyes were characterized by low toxicity in the dark and high phototoxicity under NIR light, and the most effective AmBBrI showed very low IC_50_ values in HeLa and MCF-7 cell lines [29]. The use of iodine, as in the case of symmetrical photosensitizers BDPY-DBPMB and NBDPY-DBPMB, containing a 2,6-diiodo 3,5-distyryl 1,2-bis(2-pyridylmethoxy) benzene group, is another example of such a modification of BODIPY. These compounds exhibit absorption at 671–682 nm, emission at 708–724 nm, and short-lived triplet states (0.65–1.44 µs), which enables effective singlet oxygen generation (Φ_Δ = 41–51%) in photodynamic cancer therapy. In addition, these molecules can act as selective Hg^2+^ and Fe^2+^ ion sensors in the PET mechanism, which broadens their potential applications in fluorescence bioimaging and the design of functional phototherapeutic nanoplatforms [30]. This strategy was also used in the SBOP-Lyso structure, where sulfur and iodine atoms were introduced, enabling high singlet oxygen (^1^O_2_) generation efficiency of 44.1% upon irradiation with NIR light (λ = 660 nm). The use of the M1 target group (1-(2-morpholinoethyl)-1H-indole-3-carbaldehyde) allowed for effective targeting of the molecule to acidic organelles, which was confirmed by colocalization tests (R = 0.93). SBOP-Lyso exhibits a favorable pharmacological profile, including good biocompatibility, rapid penetration into cells and model organisms, low cytotoxicity in the dark, and high photodynamic efficiency (IC_50_ = 0.2 μM). Importantly, this compound not only induces ^1^O_2_-dependent cell death, but also exhibits the ability to inhibit cancer cell migration [31].

Alternative strategies for designing PSs with high ISC efficiency that do not require the presence of heavy atoms are also being sought. One such strategy is the creation of dyads, e.g., BODIPY–acridine, where ROS generation occurs due to the formation of excimeric states. These compounds combine high fluorescence brightness with significant phototoxicity, enabling simultaneous diagnostic and therapeutic use [32]. A similar effect is achieved by covalently linking BODIPY dimers. Steric modifications and functionalization with groups that increase hydrophilicity and biorecognition enable selective action of the photosensitizer with minimal toxicity in the dark, while maintaining fluorescence in the NIR range and high singlet oxygen generation efficiency [33]. Another innovative approach is the use of *n*–π spatial coupling, which allows triplet states to be modulated without the need to further expand the π system. This strategy allows for precise tuning of the ^3^PS• level and prolonging the lifetime of the triplet state, which promotes effective triplet–triplet annihilation processes and improves the overall photoreactivity of BODIPY systems. In the case of the BDP-2 molecule, it has been shown that thanks to *n*-π modulation, the ^3^PS• energy drops to 1.34 eV and the triplet lifetime reaches 155 µs, enabling efficient energy transfer from the NIR-active PtTNP photosensitizer. This allowed for the first BODI-PY-based triplet fusion upconversion activated by near-infrared light, with high upconversion efficiency (13.3%) and preserved stability in the solid state even at very low NIR power density [34].

An interesting direction is the design of single molecules capable of simultaneous photodynamic and photothermal therapy (PTT-PDT). An example is the design of 1,7-di-tert-butyl-substituted aza-BODIPY (tBu-azaBDP), where a more twisted conformation (compared to classic Ph-azaBDP) facilitates non-radiative decay processes. Although the low-barrier rotation of -tBu groups reduces the quantum efficiency of fluorescence, it significantly improves photothermal conversion efficiency and singlet oxygen generation, increasing their potential for combined photothermal-photodynamic therapy (PTT-PDT) [35]. A similar PTT-PDT combination effect was demonstrated by the Lyso-BDP photosensitizer, which also enables fluorescence imaging. A key structural element of this molecule was the introduction of a morpholine group directing the photosensitizer to lysosomes, which allows for an increase in the local concentration of reactive oxygen species (ROS) and the effectiveness of cell death induction. In addition, the use of the N,N-diethyl-4-vinylaniline fragment enabled red-shift absorption and emission, which increases the compatibility of Lyso-BDP with deep penetration of NIR light into tissues. As a result of NIR irradiation, Lyso-BDP not only generates singlet oxygen (^1^O_2_), which determines the effect of PDT, but also induces a local thermal effect leading to protein denaturation and cell membrane damage. The synergy of both mechanisms significantly increases anticancer efficacy and overcomes the limitations associated with hypoxia and the resistance of cancer cells to PDT monotherapy [36].

**Figure 3:**
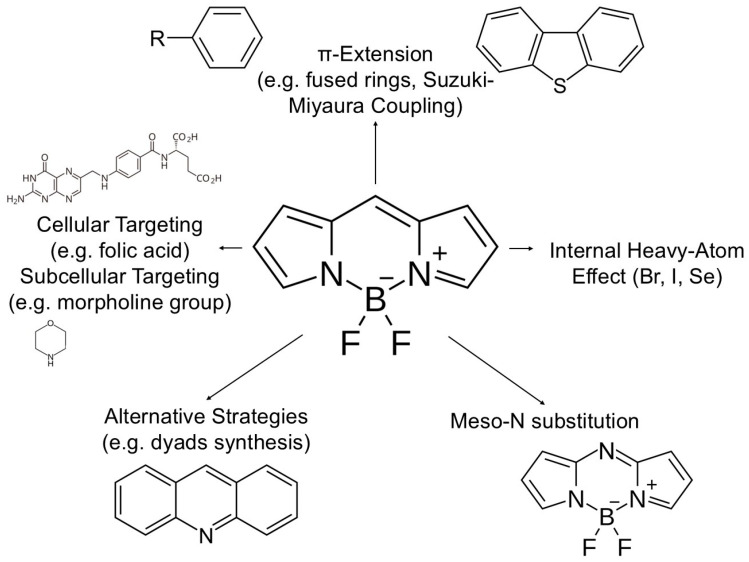
Hierarchical molecular design strategies for optimizing BODIPY photosensitizers. The central diagram illustrates the structure-property relationships used to tune the BODIPY core for theranostic applications. (i) π -Extension: Fusion of aromatic rings (e.g., benzo/thieno-fusion) reduces the HOMO-LUMO gap, red-shifting absorption into the NIR range. (ii) Heavy-Atom Effect: Introduction of Iodine (I) or Bromine (Br) at the 2,6-positions enhances spin–orbit coupling, thereby increasing the intersystem crossing (ISC) rate and singlet oxygen quantum yield (ΦΔ). (iii) Heavy-Atom-Free Approaches: Utilization of orthogonal donor-acceptor dimers or spin-converters to promote ISC without toxic heavy metals. (iv) Targeted Functionalization: Conjugation with ligands (e.g., biotin, RGD peptides) or organelle-targeting groups (e.g., TPP for mitochondria) to ensure precise subcellular accumulation and reduce off-target toxicity. (v) Core Modification: Substitution of the meso-carbon with nitrogen to form the aza-BODIPY core, significantly red-shifting absorption (Created by the authors).

It should be noted that the chemical strategies described are often combined with the implementation of targeting groups, which allows for precise subcellular localization. Examples include targeting lysosomes (Lyso-BDP [36], SBOP-Lyso [31]) or mitochondria (AmBXI [29]), which further intensifies the desired therapeutic effect. In order to achieve optimal photophysical and therapeutic properties of BODIPY photosensitizers, scientists have developed a number of rational molecular design strategies. The diagram below visualizes the main synthetic and functionalization approaches that allow for precise adaptation of BODIPY molecules to the requirements of advanced phototherapy (Figure 3).

Synthesizing these approaches, a clear trade-off emerges. The heavy-atom effect (iodination/bromination) remains the most direct and synthetically accessible route to high singlet oxygen yields (ΦΔ > 0.5$). However, it often suffers from increased dark toxicity and short triplet lifetimes due to the energy gap law. In contrast, heavy-atom-free strategies (e.g., radical dimerization, twisted donor-acceptor systems) offer superior biocompatibility and tunable lifetimes but generally require more complex synthetic architectures to achieve comparable ROS generation efficiency. Consequently, heavy-atom strategies currently dominate acute therapeutic applications, while heavy-atom-free designs are gaining traction for sensitive, long-circulation nanoplatforms. To illustrate the practical dimension of the design strategies discussed above, Table 1 summarizes the key photophysical parameters of selected BODIPY derivatives and nanotechnology systems based on them. The data presented highlight the direct impact of structural modifications—such as the expansion of the π system or the introduction of electron-donating substituents—on the shift in spectral bands to the desired NIR range and on the efficiency of radiative processes, which determine their potential in imaging and therapy.

## 5. Theranostic Nanoplatforms Based on BODIPY Photosensitizers

In recent years, phototheranostic nanoplatforms based on BODIPY dyes have been developed, integrating diagnostic and therapeutic functions. Research on BODIPY-based nanophotosensitizers reveals that the process of nanostructuring significantly increases their usefulness in photodynamic and photothermal therapy of cancer. The formation of nanoparticles through dye precipitation, supramolecular interactions, or polymer encapsulation leads to improved solubility in aqueous environments, increased biocompatibility, and more effective accumulation in cancerous tissues due to the EPR effect and receptor-mediated endocytosis. Of particular interest are systems with NIR absorption (800–900 nm), which are characterized by high singlet oxygen generation efficiency (30–60%) and in vivo efficacy. In addition, nano-BODIPYs with increased non-radiative transition rates have been developed, enabling effective conversion of energy into heat in PTT, as well as systems combining PDT/PTT mechanisms, allowing the limitations resulting from tumor hypoxia to be overcome [41].

### 5.1. Nanoformulations and Self-Organizing Systems

The development of BODIPY-based nanoplatforms typically begins with strategies to improve solubility and bioavailability through nanostructurization. Depending on the molecular design and carrier material, these systems can be broadly categorized into self-assembling amphiphilic systems, polymer-encapsulated micelles, and lipid or polypeptide-based carriers.

#### 5.1.1. Amphiphilic and Self-Assembling Systemst

One of the most direct approaches to creating nanotheranostics is the design of amphiphilic dyes capable of self-assembly. For instance, amphiphilic BODIPY-benzothiadiazole nanoparticles (BDP-BT NPs) have been developed, which, thanks to PEG modification, exhibit increased water solubility and biocompatibility. These nanoparticles generate reactive oxygen species and local hyperthermia after irradiation with NIR light, enabling synergistic photodynamic and photothermal therapy [42]. Similarly, structural twisting can be utilized to drive assembly and photophysical properties. The use of B,O chelation and a julolidine segment results in aza-BODIPY modifications that lead to a strong twisting of the molecule structure, promoting increased inter-system crossing efficiency. The resulting ROBDP dye forms stable, self-organizing nanoparticles capable of simultaneous photodynamic and photothermal action, inducing both late apoptosis and necrosis in ovarian cancer models [43].

#### 5.1.2. Polymeric Encapsulation and Micelles

To further enhance stability and circulation time, BODIPY derivatives are frequently encapsulated within biocompatible polymers. Diiodo-substituted BODIPY with NIR absorption and emission properties (SNBDP) was used to prepare nanoparticles (SNBDP NPs) by nanoprecipitation using poloxamer. These nanoparticles are characterized by high stability, effective singlet oxygen generation (ΦΔ = 40%), and the ability to image cancer cells [44]. More complex supramolecular interactions have also been explored, such as the PEG2000-IR806/BODIPY (PIBY NPs) system, obtained through the co-self-assembly of an amphiphilic and hydrophobic dye. The resulting rod-shaped nanoparticles exhibited emission in the NIR-II range and high photothermal conversion efficiency (38.5%) [45]. Furthermore, block copolymers allow for precise control over nanoparticle size. AZB-I@PEG-b-PCL nanoparticles, containing an iodinated aza-BODIPY encapsulated in a PEG-b-PCL copolymer, exhibited high colloidal stability and fluorescence emission in the NIR range. In vivo studies confirmed 49.8% inhibition of tumor growth with no significant systemic toxicity [46]. A similar PEGylated strategy was applied to BODIPY-Br2 (PEG-BDP), which showed high efficiency in ROS generation and significant accumulation in tumor tissues [47].

#### 5.1.3. Lipid and Supramolecular Architectures

Beyond synthetic polymers, lipid and peptide-based systems offer improved biocompatibility and biomimetic properties. For example, DBNPs nanoparticles were formed as a result of supramolecular co-assembly of a donor–π–acceptor BODIPY with phosphocholine lipids. These particles exhibited high photothermal conversion efficiency (η = 37.6%) and induced a synergistic PDT/PTT effect under 808 nm irradiation [48]. In the realm of peptide-based carriers, an iodinated NH_2_-BDPI derivative was encapsulated in an amphiphilic polypeptide to form nano-NH_2_-BDPI structures. This system leverages the EPR effect for passive targeting and responds to biochemical signals like H_2_S, enabling effective photodynamic ablation at low light intensities [49]. Finally, recent advances include surfactant-free approaches, such as the enhanced D-A-D structure (BDP-AP NPs). These amphiphilic nanoparticles self-assemble without additional surfactants and exhibit a high photothermal efficiency of 61.42%, representing a significant step towards simplified yet effective therapeutic nanoplatforms [50].

### 5.2. Targeted Systems

In the study by Yin et al., nine new BODIPY dyes were developed in the NIR range, in which modifications at positions 3 and 5 with an extended coupling chain increased the effectiveness of synergistic photothermal and photodynamic therapy. This was explained by the effect of extended coupling and photoinduced electron transfer (PET). Encapsulation of BDPX-M in DSPE-PEG2000-RGD nanoparticles and lecithin ensured good water solubility, biological stability, and high photothermal conversion efficiency (42.76%), enabling effective single-wave PTT/PDT treatment of tumors [51]. A new example of nanoparticles based on BODIPY dyes are thiophene-conjugated lactose-functionalized derivatives, which exhibit strong absorption in the NIR range (640–674 nm) and the ability to self-assemble into homogeneous nanostructures with a diameter of approximately 30 nm. The introduction of bromine atoms into the BODIPY core significantly increases the singlet oxygen generation efficiency (ΦΔ = 0.47), making the PBrTB derivative one of the most promising photosensitizers in this group. The presence of lactose enables the recognition of ASGP receptors on hepatocarcinoma cells, increasing their cellular uptake and therapeutic selectivity [52]. An innovative approach to increasing the efficiency of BODIPY photosensitizers in the NIR range is the use of the resonance energy transfer (RET) mechanism in the construction of dyadic systems. In this strategy, the energy donor (distyryl-BODIPY) was coupled with an iodinated derivative of distyryl-BODIPY acting as the actual photosensitizer, creating a RET-BDP system with significantly increased absorption and singlet oxygen generation efficiency in the NIR range. After encapsulation in the biodegradable copolymer F-127-FA, homogeneous, water-dispersible nanoparticles with cancer cell targeting properties were formed. RET-BDP showed significantly increased photodynamic efficacy both in vitro and in vivo, even when using a low-power NIR source, highlighting the potential of this platform as an efficient new-generation NIR nanophotosensitizer [53]. Other nanoparticles, Apt-TNP, have been functionalized with the Apt S1 aptamer, which ensures high specificity towards cancer cells. The BDP-688 probe based on the BODIPY fluorophore contained in their structure exhibits fluorescence activation in the acidic lysosomal environment, enabling precise localization of cancer cells and monitoring of the course of therapy. At the same time, the NIR-absorbing photosensitizer R16FP generates reactive oxygen species (ROS), which induce lysosomal membrane permeabilization (LMP), leading to cell death via a cathepsin-dependent pathway. Importantly, the Apt-TNP system allows real-time monitoring of the therapeutic effect, which is a significant step towards personalized PDT and minimization of side effects [54]. Another example is the FMAB NPs system based on the aza-BODIPY (MeOABBr) photosensitizer, which, thanks to its strong absorption in the NIR range, exhibits high reactive oxygen species generation efficiency (ΦΔ = 84%) and photothermal properties. Functionalization of nanoparticles with folic acid (FA) ligand and triphenylphosphonium (TPP) cation enables their dual targeting of cancer cells and mitochondria, which increases the selectivity of therapy and intensifies the apoptotic effect. Under the influence of NIR radiation, FMAB NPs cause simultaneous oxidative damage and mitochondrial hyperthermia, leading to an integrated PDT/PTT effect. At the same time, this system acts as a theranostic agent, enabling multimodal tumor imaging using fluorescence, photoacoustic, and photothermal methods. The results of the study confirm the high anticancer efficacy of FMAB NPs with limited toxicity to healthy tissues [55]. In turn, the use of BODIPY-Br_2_ in galactose-targeted polypeptide microspheres enables simultaneous NIR fluorescence imaging and selective photodynamic therapy of cancer cells. Amphiphilic microspheres act as biodegradable carriers, increasing the accumulation of photosensitizer in cells with galactose receptors, which allows for effective cytotoxicity at minimum light energy density [56]. Other BODIPY nanoparticles with a donor-π-acceptor (D-π-A) structure were also developed with thiophene modification and iodine atoms, exhibiting simultaneous high photodynamic and photothermal activity. BDP4 NPs generated ROS types I and II and exhibited photothermal conversion at a level of 44%. Folate modification of the nanoparticles enabled selective accumulation in the tumor, and a single injection combined with 808 nm irradiation resulted in complete tumor elimination and metastasis inhibition, confirming the potential of these nanoplatforms in the treatment of triple-negative cancers [57]. Modern strategies to increase the effectiveness of photodynamic therapy (PDT) increasingly use supramolecular systems that improve the solubility, stability, and selectivity of photosensitizers. An example of this approach is the PS3⊂WP5 nanosystem, composed of the photosensitizer BODIPY (PS3) and mannosylated pillar[5]arene (WP5). The formation of a host-guest complex increases the hydrophilicity of the PS3 molecule and allows it to self-assemble into stable nanoparticles. The PS3⊂WP5 system exhibits strong absorption in the near-infrared (633 nm) and very high singlet oxygen generation efficiency (ΦΔ = 0.95), which translates into high photodynamic activity. The presence of mannose residues enables selective targeting of cancer cells through interaction with mannose receptors, increasing cellular uptake [58].

### 5.3. Smart Systems

Redox-sensitive PEG-SS-BDP nanoparticles (PSSBDP NPs) were developed, in which brominated BODIPY was linked to a PEG block via a disulfide bond. This system enables selective release of the photosensitizer in cancer cells in response to excess glutathione, ensuring local singlet oxygen generation, apoptosis induction, and high photodynamic therapy efficacy. The nanoparticles also exhibit NIR imaging capability, allowing for monitoring of tumor accumulation and image-guided therapy [59]. BODIPY nanoparticles (BDPmPh, BDPbiPh, BDPtriPh) with tunable NIR light penetration depth have been developed, with diethylamine groups that react to the low pH of lysosomes. BDPtriPh NPs exhibit high photothermal efficiency (60.5%) and strong cytotoxicity towards cancer cells at minimal doses, as well as effective accumulation in tumors due to the EPR effect. This system enables pH-dependent, synergistic photodynamic and photothermal therapy while limiting side effects on healthy tissues [60]. Similar properties are exhibited by the LipoHPM smart nanoplatform [61]. Other pH-sensitive NAB NPs additionally allow for real-time photoacoustic and photothermal imaging [62].

### 5.4. Photoimmunotherapy Systems

An example of a modern multifunctional nanoplatform is BDP-6@F127, in which formylated Aza-BODIPY absorbing in the NIR range has been encapsulated in the amphiphilic copolymer Pluronic F-127. This system exhibits simultaneous photodynamic (type I and II) and photothermal effects, generating large amounts of ROS even under hypoxic conditions. BDP-6@ F127 nanoparticles are characterized by high biocompatibility, effective induction of cell apoptosis, and the ability to activate an immune response when combined with checkpoint blockade therapy (ICB), resulting in an enhanced abscopal effect and reprogramming of the immunosuppressive tumor microenvironment [41]. Another example is self-assembling mt-NPBodipy, composed of the cationic polymer Pmt and the biodegradable PBodipy polymer containing the photosensitizer BODIPY. This design allows simultaneous targeting of tumor cell membranes and intense ROS generation under NIR radiation, leading to destabilization of lipid structures and induction of cell death. In addition, the induced local lipid peroxidation initiates an immune response [63]. An example of this approach are also PPNP polymer nanoparticles capable of simultaneous photodynamic activation and siRNA delivery. Under the influence of NIR-II radiation, these nanoparticles induce a cascade of immunogenic cell death and simultaneously release siRNA against PD-L1 in a glutathione-dependent manner, leading to the reversal of tumor immunosuppression. The synergistic effect obtained results in significant tumor growth inhibition and enhanced immune memory, indicating the high therapeutic potential of platforms integrating NIR-II PDT with gene therapy [64]. Similarly, the B5@HMON nanoplatform, in which the bithiophene aza-BODIPY dye was loaded into a porous organosilicate HMON structure containing disulfide bonds. The use of HMON allowed for an increase in the solubility of B5, its selective activation in a GSH-rich environment, and enhancement of ROS generation and the photothermal effect. Under a single light source (808 nm), this nanoplatform induces immunogenic cell death, leading to calreticulin translocation, dendritic cell maturation, and enhanced CD8^+^ lymphocyte infiltration. In vivo models have shown almost complete regression of 4T1 tumors [65]. An innovative solution in the field of immunological phototherapy is the NPPDT@CXB system, based on a biodegradable polymer containing BODIPY (PPDT) monomers. After encapsulation of the cyclooxygenase-2 inhibitor (celecoxib), a system combining the functions of a photosensitizer and an immunomodulatory agent was obtained. When exposed to 808 nm light, NPPDT@CXB generates ROS, inducing immunogenic cell death (ICD) and triggering an antitumor response, while simultaneously releasing CXB, which inhibits the COX-2/PGE_2_ pathway and reduces PD-L1 expression in tumor cells. As a result, the system leads to significant suppression of primary tumor growth and metastasis [66].

### 5.5. Systems That Overcome Hypoxia

In recent years, strategies enabling the continuous generation of singlet oxygen (^1^O_2_) in photodynamic therapy have attracted particular interest, especially in conditions of tumor hypoxia and limited light access. An example of such an approach is the development of aza-BODIPY BDY functionalized with a 2-pyridone group, which enables so-called sustainable photodynamic therapy. The BDY dye, which absorbs in the NIR range (maximum 586 nm in CH_2_Cl_2_), has been converted into BDY NPs nanoparticles, characterized by a broad absorption band (530–680 nm) and a red shift in an aqueous environment. Thanks to the presence of the 2-pyridone group, BDY undergoes a reversible transformation into the endoperoxide form (BDY-EPO), which spontaneously releases ^1^O_2_ even in the dark, ensuring continuous photodynamic activity without the need for constant illumination. Furthermore, BDY NPs exhibit high photothermal conversion efficiency (35.7%), strong photoacoustic signals (PAI), and good biocompatibility and photochemical stability. In vitro and in vivo studies (on the HeLa model) reported significant tumor growth inhibition (93.4%) after synergistic PDT/PTT [38]. P-Hb-B polypeptide nanoparticles were developed, in which hemoglobin acts as an oxygen carrier and BODIPY-Br_2_ enables ROS generation and NIR fluorescence imaging. This system effectively increases the efficiency of photodynamic cancer therapy under hypoxic conditions, enables real-time tracking of nanoparticles, and achieves high cytotoxicity at low irradiation energy [67]. The development of a protein nanoplatform using the I-BODIPY photosensitizer, which enables simultaneous NIR imaging and effective photodynamic eradication of cancer cells, has also been described. A key element in the design of these nanoparticles was the inclusion of catalase, which ensures local oxygen generation and thus compensates for the hypoxia characteristic of the tumor microenvironment. The use of multipoint supramolecular interactions allowed for the creation of stable, self-organizing structures with increased efficiency in generating reactive oxygen species, which translated into increased PDT efficacy in in vitro and in vivo models [68]. A DF-BODIPY@ZIF-8 nanocomposite was also developed, in which the DF-BODIPY photosensitizer was loaded into a ZIF-8 metal–organic framework, enabling oxygen generation in the tumor. The increase in the electronegativity of the substituents reduces the energy difference between the singlet and triplet states, increasing the efficiency of singlet oxygen generation. This system exhibits high cytotoxicity towards cancer cells in vitro and effectively eliminates 4T1 tumors in vivo, overcoming the limitations of hypoxia and increasing the effectiveness of PDT [69]. From a clinical perspective, the discussed strategies offer distinct advantages and limitations. Oxygen-carrying systems (e.g., hemoglobin-based) are conceptually simple but limited by their loading capacity and premature oxygen release. In situ oxygen generators (e.g., MnO_2_, catalase) are smarter, responding to the TME, but their efficacy depends on endogenous H_2_O_2_ levels, which can vary. Currently, the most robust approach appears to be the shift towards Type I photosensitizers or sustainable release systems (like endoperoxides), as they bypass the reliance on external oxygen supply entirely, thereby addressing the root cause of PDT resistance in deep, hypoxic tumors rather than merely treating the symptom.

### 5.6. Drug-Related Systems

Hybrid structures integrating phototherapy with chemotherapy, which exhibit a synergistic effect, are also gaining increasing attention. Despite significant progress, challenges include improving cellular uptake, reducing heavy metal toxicity, stabilizing dye loading in nanocarriers, and developing nanosystems that respond to specific tumor microenvironment markers [70]. An example of this approach is the structurally complex FBD-M derivative, in which the BODIPY core acts as a photosensitizer and NIR fluorophore. Rational modification of the molecule by attaching a pentafluorobenzene group gave it the ability to transport oxygen and thus alleviate hypoxia in the tumor microenvironment, which significantly increases the effectiveness of photodynamic therapy. The presence of the morpholino group enabled the selective targeting of FBD-M to the lysosomes of neoplastic cells, while the introduction of nitrogen mustard gave it the cytotoxic properties characteristic of alkylating chemotherapeutics. The synergy of photodynamic, photothermal, and chemical mechanisms results in increased anticancer efficacy in cell and animal models, even under hypoxic conditions. These results prove that FBD-M represents a new generation of multifunctional phototherapeutic BODIPY systems with clinical potential [39]. Another example of a nanoplatform designed to overcome tumor hypoxia is the MDSP NP system, in which a specially designed aza-BODIPY with strong absorption in the NIR range (~853 nm) has been co-loaded with doxorubicin on MnO_2_ nanoparticles with a characteristic hydrangea structure. These nanoparticles undergo rapid degradation in the acidic and hydrogen peroxide-rich microenvironment of the tumor, leading to oxygen release and a significant reduction in hypoxia. This process promotes both increased ROS generation under PDT conditions and more effective drug release. In addition, MDSP NPs exhibit a strong photothermal effect, which facilitates the uptake of the nanopreparation and enhances cytotoxicity. The result is a synergistic therapeutic response combining chemotherapy, PDT, and PTT, confirmed in in vitro and in vivo studies, which highlights the high potential of this platform in the treatment of hypoxic tumors [71]. Other amphiphilic BODIPY nanoparticles (NBDP) with encapsulated doxorubicin, a two-step drug delivery system for synergistic chemo-photodynamic therapy, were described in a study by Zhang et al. The introduction of hydrophilic chains and iodine atoms enabled water solubility, shifted absorption to the NIR range, and increased singlet oxygen generation efficiency (ΦΔ = 71.1%). This system allows for simultaneous NIR imaging, ROS generation, and chemotherapeutic action, effectively overcoming the limitations of traditional phototherapy [72]. Other nanoplatforms have used the chemotherapeutic agent vadimezan, which is released in the acidic microenvironment of the tumor, leading to an antiangiogenic effect and the destruction of pathological vessels, thereby blocking the formation and development of metastases [73]. An example of a similar approach is the PolyBodipy (NP2) nanoparticle platform. This system represents a new class of BODIPY polymer photosensitizers capable of emitting in the NIR-II range. NP2 nanoparticles have the ability to initiate type II immunogenic cell death (ICD)—a form of cell death that induces strong activation of the immune system. The synergy of chemical and photodynamic interactions leads to the release of DAMPs (damage-associated molecular patterns), the maturation of dendritic cells, and the activation of cytotoxic T lymphocytes. Importantly, the NP2 platform is characterized by light-controlled activation, low toxicity in the dark, and high selectivity for the tumor microenvironment. In triple-negative breast cancer (TNBC) models, significant tumor growth suppression and increased CD8^+^ lymphocyte infiltration were observed, confirming its potential as an innovative platform combining PDT with cancer immunotherapy [74]. In another study, multifunctional BODIPY-Platinum conjugates were developed. The incorporation of platinum into the BODIPY core increases the generation of reactive oxygen species during PDT through the heavy atom effect, while maintaining cytotoxicity comparable to cisplatin. The conjugates show selective accumulation in the mitochondria of cancer cells and enable simultaneous monitoring of the location and effectiveness of therapy in vivo [75].

An interesting example of a hybrid therapeutic system is a lipid nanoplatform based on iodinated aza-BODIPY (AZB-I), in which cannabidiol (CBD) acts both as a lipid phase and as an agent enhancing the cytotoxic effect. The resulting AZB-I@Lec-T@CBD nanoparticles are characterized by high colloidal stability and significantly increased photodynamic activity. After irradiation with 660 nm light, they exhibit more than twice lower IC_50_ (4.3 nM) compared to a similar system without CBD, which results from the synergy between ROS generation (type I and II mechanisms) and CBD-induced susceptibility of cells to oxidative stress. These nanoparticles cause an enhanced apoptotic effect in HepG2 cells and a reduction in the expression of antioxidant system genes, confirming their high efficacy in liver cancer models [76]. In a similar approach, DAB NPs combine aza-BODIPY with doxorubicin in a PEG matrix using Schiff bonds, enabling pH-sensitive drug release in the tumor microenvironment. This system exhibits high ROS generation, photothermal effects, and effective accumulation in the tumor, enabling synergistic photodynamic, photothermal, and chemotherapeutic therapy with simultaneous NIR imaging and minimal side effects [77].

### 5.7. Other Strategies and Carrier Platforms

An interesting approach to increasing the bioavailability and effectiveness of BODIPY photosensitizers is their complexation with serum proteins. It has been shown that diiodinated BODIPY derivatives form stable complexes with human serum albumin (HSA), stabilized mainly by van der Waals interactions in the IB subdomain of the protein. The formed BODIPY/HSA complexes are characterized by increased solubility in an aqueous environment and increased singlet oxygen generation efficiency, which directly translates into increased photodynamic efficiency. This phenomenon indicates that the use of HSA as a natural carrier may be an effective strategy for improving the biocompatibility and phototherapeutic activity of BODIPY dyes in cancer treatment [78]. Organic nanoparticles based on aza-BODIPY derivatives (TAS-BP, TES-BP, TESO-BP) have also been developed, which, thanks to their donor-acceptor-donor structure and coupling with TPA or TPE, enable the simultaneous generation of reactive oxygen species and local heat after irradiation with NIR light (808 nm). Nanoparticles exhibit high photothermal efficiency (53–66%) and significant cytotoxicity towards cancer cells while maintaining good biocompatibility, making them promising candidates for synergistic photodynamic and photothermal therapy [79]. An interesting solution is the use of soluble microneedles as carriers for aza-BODIPY photosensitizers. The AZP10 used, characterized by high reactive oxygen species generation efficiency (ΦROS ≈ 0.62), showed strong cytotoxicity against squamous cell carcinoma cells of the oral cavity after irradiation with 670 nm light. The placement of AZP10 in the micro-puncture structure enabled a more than ninefold increase in transdermal flux and long-term retention of the photosensitizer at the application site, which significantly improved the therapeutic effect. In a mouse model of oral cancer, the use of three cycles of PDT with microneedles led to almost complete tumor regression, comparable to the result after intracellular administration of the photosensitizer [80]. Another example of a hybrid nanoplatform using BODIPY dyes are MTAB nanoparticles, composed of mesoporous black titanium(IV) oxide (TiO_2_) functionalized with an aza-BODIPY probe. This structure combines the photocatalytic properties of TiO_2_ with the ability to absorb radiation in the NIR range, characteristic of aza-BODIPY dyes. The integration of both components leads to the formation of a system with ordered energy levels, enabling effective separation of photogenerated electron-hole pairs and limiting the recombination of charge carriers. As a result, the MTAB nanoplatform generates high amounts of reactive oxygen species (ROS) under the influence of a single NIR light source, enabling simultaneous dynamic phototherapy (PDT) and photothermal therapy (PTT). In vitro and in vivo studies have shown that MTAB effectively eliminates cancer cells and causes tumor regression [81]. BO-DIPY-cyclodextrin (BODIPY-CDs) complexes have been developed that exhibit high water solubility and biocompatibility while maintaining efficacy in photodynamic therapy. The integration of BODIPY with cyclodextrin via triazole units caused a shift in fluorescence emission towards NIR wavelengths, which enables more effective generation of reactive oxygen species and reduction in cancer cell viability. The BODIPY-β-CD complex is particularly notable for its high PDT activity against HeLa cells with minimal in vitro toxicity [82]. The photophysical properties, strategies, and results of the therapy are presented in Table 2.

## 6. The Use of PS BODIPY NIR in Antibacterial Therapy and Biofilm Control

The increasing multidrug resistance (MDR) of bacterial pathogens and the difficulties in treating biofilm-associated infections are among the most serious challenges facing modern medicine. In response to this crisis, an innovative approach is the use of phototherapy with NIR-absorbing BODIPY photosensitizers. These strategies are being developed in two main, complementary directions. The first involves the direct eradication of bacterial cells, often through a synergistic combination of photodynamic (PDT) and photothermal (PTT) mechanisms. The second focuses on the destruction of the protective extracellular matrix of the biofilm, which does not kill the bacteria directly, but weakens their mechanical barrier and significantly increases their sensitivity to conventional antibiotics [83,84].

An example of an approach aimed at destroying the biofilm matrix are activatable iodinated BODIPY derivatives (THQ-C and DMA), which exhibit high selectivity towards amyloid structures in the extracellular matrix of the biofilm. After binding to amyloids, these photosensitizers are activated, which manifests itself in increased emission in the near-infrared range and increased generation of reactive oxygen species. Photodynamic degradation of the biofilm matrix leads to a weakening of the mechanical barrier that hinders drug penetration, which in turn increases the sensitivity of the biofilm to antibiotics. Importantly, this system does not exhibit cytotoxicity towards the bacteria themselves, acting selectively on the structural components of the biofilm [85].

The second, more commonly used approach is to design systems with direct bactericidal action, often using synergistic phototherapy mechanisms. It has been shown that even simple derivatives, such as aza-BODIPY 5, exhibit significant activity against a broad spectrum of MRSA strains, including methicillin-resistant and clinical strains, with a minimum bactericidal concentration of 12.5–25 µM. The effectiveness of this photosensitizer was significantly higher than that of methylene blue, indicating its potential as a new antibacterial agent in NIR-based photodynamic therapy [86]. In order to increase its effectiveness, subsequent studies focused on nanoformulations with dual PDT/PTT action. An example is asymmetric aza-BODIPY (ABDP) in the form of nanoparticles (ABDP NPs), which are characterized by extended absorption in the near-infrared range (NIR, 639–780 nm). Encapsulation of ABDP in the biocompatible polymer Chol-PEG improves its stability in an aqueous environment and enables effective delivery to the site of infection. Under the influence of 808 nm wavelength radiation, ABDP NPs exhibit synergistic photodynamic and photothermal effects, generating reactive oxygen species (^1^O_2_) and inducing local temperature increases. This leads to the effective eradication of both Gram-positive (*S. aureus*, MRSA) and Gram-negative (*E. coli*) bacteria. Importantly, their high efficacy has been confirmed in a model of infected wounds in mice, where the use of ABDP NPs in combination with NIR irradiation accelerated the re-epithelialization process and reduced excessive inflammatory response [87]. In a study by Yu et al., it was found that Aza-BODIPY modified by “molecular surgery” exhibit significantly increased photothermal and photodynamic activity, making them effective photosensitizers in combating antibiotic-resistant bacteria. When excited by 660 nm light, the nanoparticles exhibit high photothermal energy conversion and intense production of reactive oxygen species, leading to damage to bacterial membranes and complete eradication of the infection [88]. Another advanced synergistic strategy is to combine ROS generation with controlled release of nitric oxide (NO), which has a bactericidal effect. The PNIR-II compound was constructed in a donor-acceptor system by combining thiophene with DPP and BODIPY units, which made it possible to increase the efficiency of intersystem transitions and reduce the undesirable photothermal effect, promoting stable ROS generation. After combining it with a polymeric nitric oxide (NO) donor, nanoparticles capable of selective degradation in a biofilm environment with elevated glutathione levels were obtained. Importantly, PNIR-II retains approximately 50% of its photodynamic efficacy even after passing through a 2.6 cm thick tissue barrier, making it one of the most effective NIR-II polymeric photosensitizers described to date for the eradication of deep tissue biofilms, including biofilms of multidrug-resistant Staphylococcus aureus strains [89].

## 7. Challenges and Prospects

Despite the enormous progress in the design of NIR-absorbing BODIPY photosensitizers documented in this work, a number of challenges remain to be overcome on the path to their successful clinical translation. At the same time, emerging research strategies are opening up new and promising horizons for applications.

### 7.1. Challenges in Clinical Translation

The main obstacle remains the so-called “valley of death” in drug development, i.e., the gap between promising results in in vitro and in vivo models and effective use in patients [90]. This is especially true for complex nanoplatforms [91]. Furthermore, although many of the systems described show low toxicity in the dark, there is a lack of data on their long-term accumulation in organs (e.g., the liver and spleen) and the potential toxicity associated with the degradation of polymer, lipid, or inorganic carriers (such as TiO_2_ [81] or ZIF-8 [69]). This requires further detailed toxicological studies [92]. The synthesis of many advanced BODIPY molecules and their formulation into precise nanoparticles (e.g., with controlled size and drug load) is often multi-step, costly, and difficult to scale up. Ensuring batch-to-batch consistency is a critical regulatory requirement that poses a challenge, especially for self-assembling systems [43,50,63]. In addition, many nanoplatforms base their passive targeting on the enhanced permeability and retention (EPR) effect [49,60]. However, growing criticism indicates that this effect, although pronounced in mouse models, is much less predictable and pronounced in humans. This calls into question the effectiveness of passive targeting and reinforces the importance of active targeting [93,94].

Furthermore, the interpretation of current progress must account for inherent methodological biases. The vast majority of reviewed studies rely on subcutaneous xenograft models, which, while useful for preliminary screening, poorly mimic the complex stromal barriers, high interstitial pressure, and metastatic potential of human tumors. Consequently, the high tumor inhibition rates observed in these simplified models may be overestimated compared to clinical reality. Additionally, there is a notable lack of standardization in irradiation protocols (light power density, total fluence) and singlet oxygen quantum yield measurements across laboratories. This heterogeneity, combined with a likely publication bias favoriting positive outcomes, makes direct head-to-head comparisons of different BODIPY nanoplatforms challenging and underscores the need for more rigorous, standardized reporting guidelines in future research [42,44,45,46,47,48,49,50].

### 7.2. Prospects and New Directions for Research

Despite these challenges, the field of research is developing dynamically, and new strategies offer solutions to existing problems. Although much of the current work focuses on NIR-I and NIR-II [17,45,64,74], the ultimate goal is to further shift absorption and emission into the third biological window (NIR-III: 1700–2500 nm). While NIR-II (1000–1700 nm) already offers reduced scattering compared to NIR-I, the NIR-III window provides the highest theoretical signal-to-background ratio and penetration depth due to the almost total absence of tissue autofluorescence and minimized photon scattering [95,96]. Currently, this range is dominated by inorganic materials (e.g., rare-earth-doped nanoparticles), and examples of stable, organic BODIPY photosensitizers active in NIR-III are virtually non-existent. Consequently, designing BODIPY derivatives with such narrow bandgaps without compromising stability represents the next great frontier in synthetic photomedicine.

Synergy of Photo-Immunotherapy: As demonstrated, the combination of PDT/PTT with immunotherapy [41,63,64,65,66] is one of the most promising avenues. Future research will likely focus on designing systems that not only induce immunogenic cell death (ICD) but also actively modulate the tumor microenvironment, e.g., by simultaneously delivering STING agonists or checkpoint inhibitors to elicit a potent, systemic, and long-lasting antitumor effect.

Computational Design: Instead of labor-intensive syntheses and screening tests, computational chemistry and artificial intelligence will play an increasingly important role. The use of machine learning models to predict photophysical properties (absorption, ISC efficiency) based on molecular structure will enable rapid in silico design of new, highly optimized BODIPY photosensitizers [97,98].

Beyond Oncology: Success in combating resistant bacterial biofilms [85,86,87,88,89] opens the door to other applications. Of particular interest is the ability of certain BODIPY derivatives to target amyloid structures [85] in the context of neurodegenerative diseases such as Alzheimer’s disease, opening up a whole new chapter in phototherapy applications beyond oncology [99].

## 8. Conclusions

BODIPY dyes that absorb in the near-infrared (NIR) range have established themselves as one of the most promising and versatile molecular platforms in modern phototherapy. This review has shown that their unique photophysical properties, combined with almost unlimited possibilities for chemical modification, allow for the rational design of photosensitizers (PS) with precisely defined functions. These strategies include both tailoring the molecular core to increase the efficiency of reactive oxygen species (ROS) generation and shift absorption, as well as advanced techniques for subcellular targeting and activation in response to specific microenvironmental conditions.

The evolution from simple molecules to complex theranostic nanoplatforms has revolutionized their potential, enabling the synergistic combination of photodynamic therapy (PDT) and photothermal therapy (PTT), overcoming tumor hypoxia, and effective integration with chemotherapy and immunotherapy. Furthermore, their proven effectiveness in combating antibiotic-resistant bacterial biofilms opens up entirely new therapeutic avenues beyond traditional oncology. Although significant challenges remain on the path to full clinical translation, particularly in terms of production scalability and long-term toxicology, there is no doubt that BODIPY NIR photosensitizers will form the foundation for the development of the next generation of precision, light-activated therapies.

## Figures and Tables

**Table 1:** Summary of the photophysical properties of representative BODIPY photosensitizers and nanoplatforms discussed in the paper. The absorption (λabs) and emission (λem) maxima, molar extinction coefficient (ϵ), and fluorescence quantum yield (Φf) are presented. All values refer to measurements taken at room temperature (298 K). The abbreviation “–” indicates that no data is available in the cited literature.

Dye	λabs/λem [nm]	ϵ [M^−1^ cm^−1^]	Φf	Solvent	Source
tBu-azaBDP 1	620/650	78,500	0.11	CH_2_Cl_2_	[35]
tBu-azaBDP 2	654/688	84,500	0.12	CH_2_Cl_2_	
tBu-azaBDP 3	672/715	86,000	0.09	CH_2_Cl_2_	
Ph-azaBDP 4	650/672	79,000	0.34	CH_2_Cl_2_	[37]
Ph-azaBDP 5	688/722	85,000	0.36	CH_2_Cl_2_	
BDY (2-pyridone-aza-BODIPY)	586/-	65,600	-	CH_2_Cl_2_	[38]
FBD-M	731/820	-	0.103 (in Ethanol) 0.0136 (in PBS)	PBS	[39]
BD-M	695/783	-	0.138 (in Ethanol)	PBS	
SBDPiR690	688/700	120,000	0.22	CHCl_3_	[28]
SBDPiR688	688/695	211,000	0.39	CHCl_3_	
SBDPiR698	698/705	146,000	0.38	CHCl_3_	
BT-[b]-fused BODIPY	647/680	145,061	-	CH_2_Cl_2_	[40]
BDP-1	523/540	84,500	1.0	Toluene	[34]
BDP-2	549/569	74,900	0.98	Toluene	
BDP-3	546/567	68,000	1.0	Toluene	
BDP-4	500/517	88,500	1.0	Toluene	
BDP-5	523/543	74,100	0.74	Toluene	
BDP-6	503/518	98,500	0.58	Toluene	
BDP-7	526/543	82,600	0.77	Toluene	
AmBHI	648/673	-	0.18 in Ethanol	Water	[29]
AmBMI	659/689	-	0.11 in Ethanol	Water	
AmBBrl	671/709	-	0.06 in Ethanol	Water	
BODIPY 8a	643/654	122,300	0.47	CHCl_3_	[32]
BODIPY 8b	633/645	119,900	0.63	CHCl_3_	
BODIPY 8c	648/664	100,100	0.38	CHCl_3_	
BODIPY 9a	674/689	107,300	0.069	CHCl_3_	
BODIPY 9b	664/678	98,200	0.091	CHCl_3_	
BODIPY 9c	681/697	98,400	0.052	CHCl_3_	

**Table 2:** Comparative summary of representative NIR-BODIPY nanoplatforms: Photophysical properties, targeting strategies, and therapeutic outcomes. ΦΔ—singlet oxygen quantum yield; IC_50_—half-maximal inhibitory concentration; η—photothermal conversion efficiency.

Nanoplatform	Core PS Type	Targeting/Strategy	Key Photophysical Data	Therapeutic Outcome	Source
**BDP-BT NPs**	BODIPY-benzothiadiazole	Passive (EPR), PEG-modified	IC_50_ = 22.17 μg/mL	Synergistic PDT/PTT; high cytotoxicity.	[42]
**SNBDP NPs**	Diiodo-BODIPY	Passive (Poloxamer)	ΦΔ = 40%	High stability; selective cancer cell destruction.	[44]
**PIBY NPs**	IR806/BODIPY assembly	Passive	IC_50_ = 3.96 µg/mL; PTT η = 38.5%	Tumor growth inhibition under 730 nm laser.	[45]
**AZB-I@PEG-b-PCL**	Iodinated Aza-BODIPY	Passive (EPR)	NIR fluorescence	49.8% tumor inhibition (Day 3); suppression up to 14 days.	[46]
**DBNPs**	Donor–π–Acceptor BODIPY	Lipid co-assembly	PTT η = 37.6%	Apoptosis induction; synergistic PDT/PTT in vivo.	[48]
**BDP-AP NPs**	D-A-D BODIPY	Surfactant-free self-assembly	PTT η = 61.42%	High antitumor efficacy with minimal toxicity.	[50]
**PBrTB**	Thiophene-lactose BODIPY	Active (Lactose -> ASGP receptor)	ΦΔ = 47%; IC_50_ = 75.8 nM or 66.4 nM	Enhanced cellular uptake in hepatocarcinoma.	[52]
**FMAB NPs**	Aza-BODIPY (MeOABBr)	Dual: Folic Acid (tumor) + TPP (mito)	ΦΔ = 84%; PTT η = 40%	Integrated PDT/PTT; mitochondrial hyperthermia.	[55]
**PS3** **⊂** **WP5**	BODIPY host-guest	Active (Mannose receptor)	ΦΔ = 95%	Very high photodynamic activity.	[58]
**BDPtriPh NPs**	BODIPY-diethylamine	pH-responsive (Lysosomes)	IC_50_ = 4.16 μM; PTT η = 60.5%	Effective tumor accumulation; synergistic therapy.	[60]
**BDY NPs**	2-pyridone func. BODIPY	Sustainable PDT (Hypoxia)	PTT η = 35.7%	93.4% tumor growth inhibition (HeLa model).	[38]
**AZP10 Microneedles**	Aza-BODIPY	Transdermal delivery	ΦΔ = 62%	Complete tumor regression in oral cancer model.	[80]

## Data Availability

No new data were created or analyzed in this study.
